# An Unusual Initial Presentation of Elderly-Onset Crohn’s Disease

**DOI:** 10.7759/cureus.10173

**Published:** 2020-08-31

**Authors:** Kenan Raddawi, Albert Fleisher, Charmian Sittambalam, Muhammad N Yousaf

**Affiliations:** 1 Internal Medicine, MedStar Franklin Square Medical Center, Baltimore, USA; 2 Geriatrics, MedStar Franklin Square Medical Center, Baltimore, USA; 3 Internal Medicine, MedStar Union Memorial Hospital, Baltimore, USA; 4 Internal Medicine, MedStar Good Samaritan Hospital, Baltimore, USA; 5 Section of Digestive Diseases, Yale University School of Medicine, New Haven, USA

**Keywords:** crohn’s disease, intestinal obstruction, inflammatory bowel disease, elderly onset crohn’s disease

## Abstract

Crohn’s disease affects individuals across all age groups. However, given that it is more prevalent in younger adults, less attention is typically paid to elderly-onset Crohn’s disease. The incidence of late-onset inflammatory bowel disease is around 8 per 100,00 patients per year in the United States. The hallmark symptoms of Crohn’s, such as abdominal pain, diarrhea, and weight loss, may be absent in elderly patients, therefore making it more challenging to reach a diagnosis and initiate treatment in a timely manner. Crohn’s disease can lead to multiple complications, including abdominal abscess, enteroenteric or perianal fistulas, and bowel obstruction. Nevertheless, it is highly uncommon to have bowel obstruction as the initial sign of the disease.

## Introduction

Inflammatory bowel disease (IBD), of which Crohn’s disease is a part of, is typically a disease of early adulthood with initial onset of symptoms in patients usually in their early 20s. However, due to the bimodal distribution of the disease, another late peak can occur between 50 and 70 years of age [1,2]. Only 10%-15% of patients diagnosed with IBD are older than 65 years of age [3]. Elderly-onset Crohn’s disease (EOCD) is defined as onset of symptoms after the age of 60 years. The estimated incidence of late-onset IBD is 4 per 100,000 patients per year (United States) and 8 per 100,000 patients per year (Europe) [4], making this a very rare finding and therefore not likely to be readily thought of as a possible diagnosis. 

Clinically, patients with EOCD could present with non-specific symptoms, such as constipation, fever, malaise, and gastrointestinal hemorrhage, without the typical symptoms that are usually seen in younger population, such as cramping abdominal pain, diarrhea, or weight loss [5]. The extraintestinal manifestations of EOCD are similar to adult population; however, there is a lower incidence of family history of IBD in older patients [6]. The location of intestinal involvement is thought to be similar to that of younger patients; however, there is paucity of data in current literature. Several characteristics of EOCD, such as atypical presentation, slow progression, and the presence of multiple comorbidities, make it more challenging to reach the diagnosis. 

## Case presentation

**Figure 1:**
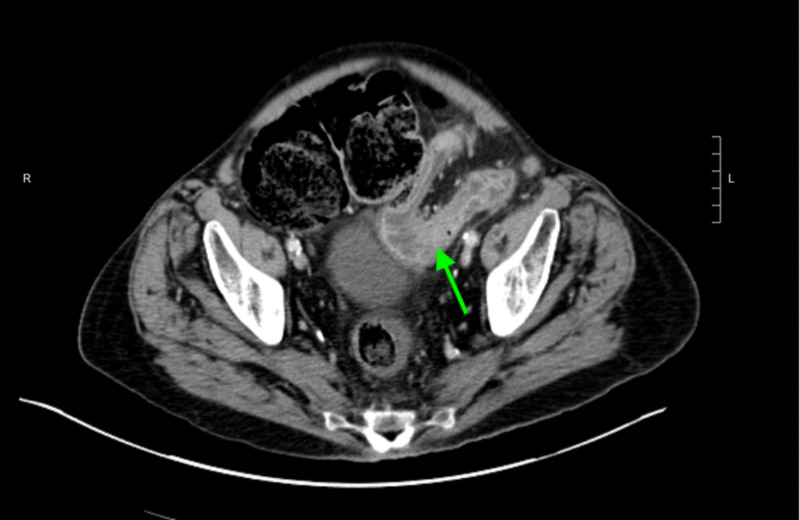
CT of the abdomen and pelvis with intravenous contrast demonstrating segmental inflammatory change involving the distal descending colon (arrow) suggestive of colitis.

A 77-year-old male with peripheral vascular disease and chronic pulmonary obstructive disease presented to the emergency room with persistent nausea and non-biliary, non-bloody emesis for one day. This was preceded by three months of poor appetite, intermittent fecal urgency, and loose stools with mucus discharge. He denied abdominal pain, fever, or weight loss. He had three routine screening colonoscopies in the past with two of them showing non-specific colitis and the most recent one, six years prior to his presentation, revealing granuloma. Despite these findings, there were no further investigations or follow-ups. At presentation, his vitals were notable for a slightly elevated blood pressure (146/85 mm Hg) and a heart rate of 88 beats per minute. Laboratory workup was significant for elevated lactic acid (2.3 mmol/L) and leukocytosis (white blood cell 15.7 k/µL, neutrophil-dominant). Physical examination revealed a severely distended tympanic abdomen without tenderness. CT scan of the abdomen and pelvis with IV contrast (Figure 1) demonstrated segmental colitis of the descending colon leading to partial mechanical obstruction and marked gaseous distention of the proximal colon. CT angiogram of the abdomen and pelvis was performed to rule out vascular etiologies and was unremarkable for vascular stenosis or occlusion.

**Figure 2:**
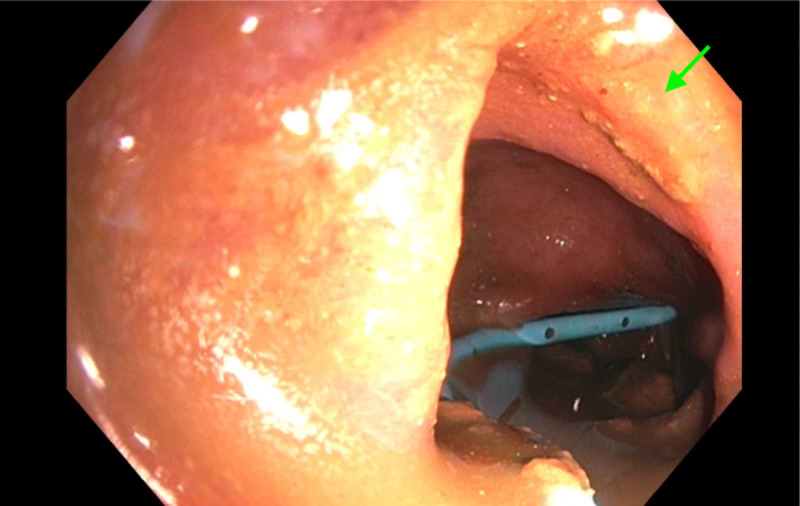
Colonoscopy showing the descending colon with segmental inflammation characterized by erythema, friability, and granularity (arrow) along with significant stricture of the descending colon with a decompression tube.

Colonoscopy (Figure 2) showed severe rectosigmoidal colitis. Tissue biopsy showed severe acute colitis with ulceration, acute cryptitis, lamina propria, and increased chronic inflammation with crypt architectural distortion. Given the patient’s current and previous historical findings, he was diagnosed with EOCD. The patient underwent a laparoscopic-assisted transverse loop colostomy; however, the obstruction remained thereafter with significant symptom burden. Therefore, per his goals of care, he was discharged home with comfort care measures. 

## Discussion

EOCD can lead to a multitude of complications, including abdominal abscesses, enteroenteric or perianal fistulas, and bowel obstruction. Intestinal obstruction usually arises from acute inflammation, adhesions, muscular hyperplasia, or recurrent inflammation and formation of fibrotic scare tissue. The likelihood of these complications increases with the duration of the disease [6,7]. The most common location of de novo strictures are the ileum and the ileocolonic regions, presumably due to the smaller diameter of the ileum relative to the colon [8,9]. This case illustrated an atypical finding of absent gastrointestinal symptoms despite clear colonoscopy and pathohistological findings suggestive of IBD that was seen years prior to his presentation with acute intestinal obstruction. We postulated that the patient’s colonic obstruction is most likely in the setting of recurrent asymptomatic colitis causing fibrotic stricture. 

Elderly patients might have mild symptoms for years before being diagnosed. The average time to establish the diagnosis is six years in the elderly as compared to two years in younger patients [10]. The differential diagnosis of abdominal symptoms in the elderly is broad. More prevalent diseases in this age group, such as colorectal malignancy, diverticular disease, and microscopic colitis, could mimic EOCD. If endoscopy and histology reveal findings compatible with granulomatous inflammation and/or ulceration, IBD/EOCD must considered. Therefore, imaging studies and colonoscopy should be readily performed with proper interpretation of results, despite absence of symptoms, to prevent significant complication in the future that could be fatal.

Management of EOCD depends on multiple factors, including severity of clinical presentation, location of intestinal involvement, extension of the disease, extraintestinal manifestation, and comorbidities. A comprehensive geriatric assessment should be done to assess for patient frailty and ability to withstand proposed treatment options that would be of benefit to the patient. Pharmacological management of younger and older patients is similar; however, use of biological agents in elderly patients is not standardized due to lack of data in this population. In patients unresponsive to the medical management or those presenting with disease complications, such as toxic megacolon, obstruction, fistula formation, or incessant hemorrhage, surgical management (bowel resection, strictureplasty, or bypass) may be required. Factors associated with poor surgical outcomes are male gender, advanced age, and low serum albumin levels [10]. Elderly patients with IBD have an increased operating room time, increased rate of postoperative complications, and increased length of hospital stay [11]. This again necessitates the need for comprehensive geriatric assessment prior to any surgical intervention to ensure the benefits of surgery will outweigh the risks. 

## Conclusions

This case study serves as a reminder of the bimodal age of onset of Crohn’s Disease. It also highlights the more subtle features of the disease in the older population, which could lead to delay in care and undesirable complications, including death. A high index of suspicion is needed for possible IBD/EOCD in elderly patient, particularly if endoscopic and histologic findings are suggestive of the disease, regardless of the absence or presence of the traditional symptoms. Only then can early management strategies be implemented to prevent further complications and potentially allow for the patient to be of better functional status to endure the proposed treatments.
